# Supplementary material for: Are carbon emissions trading and green financial instruments synergistic? -Comprehensive quantitative research based on content analysis
Source: PLoS One. 2024 Mar 7;19(3):e0298601. doi: 10.1371/journal.pone.0298601 (PMC10919601; doi:10.1371/journal.pone.0298601)
Supplement: S1 Data — (ZIP) [file pone.0298601.s001.zip › Data/地级市Z_F工作报告.zip!/░▓╗╒/╗┤─╧╩╨/└┤╫╘╣1⁄2╓┌║┼í╛┬φ┐╦╩2╛▌═°í┐_2011.docx]

各位代表：

现在，我代表市人民政府向大会报告工作，请予审议，并请市政协委员和其他列席人员提出意见。

**一、克难奋进、成果辉煌的“十一五”**

“十一五”时期，是淮南发展进程中极不平凡的五年，也是经济社会发展最好、城乡面貌变化最大、人民群众受益最多的五年。全市人民在市委的坚强领导下，全面贯彻落实科学发展观，万众一心，开拓进取，成功战胜特大自然灾害，妥善防控公共卫生事件，有效应对国际金融危机，圆满完成“十一五”目标任务，开创了淮南现代化建设新局面。

**五年来，综合实力大幅提升。**预计2005到2010年，全市生产总值从263.6亿元增加到600亿元，年均增长13.2%；财政收入从36.5亿元增加到106亿元，年均增长23.8%；粮食产量从100.5万吨增加到139.1万吨；规模以上工业增加值从116.1亿元增加到388亿元，年均增长19.2%；社会消费品零售总额从81.7亿元增加到186.5亿元，年均增长18%。累计完成固定资产投资1318.2亿元，是“十五”时期的3.9倍。规模以上工业企业从153家增加到532家。工业化率从44.8%提高到59%，城镇化率从59.1%提高到65%。

**五年来，产业结构持续优化。**围绕立足煤、延伸煤、不唯煤、超越煤，实施“T”型空间布局和产业布局。煤电产业加速提升，原煤年产量从4018万吨提高到8100万吨，年发电量从230亿千瓦时提高到465亿千瓦时，分别增长101.6%、102.2%，率先在全国建成“亿吨级”煤电基地。煤化工、煤机产业加快培育，一批重大项目相继落地。纺织服装、轻工食品等传统产业加快振兴。高新技术产业增加值年均增长28%。现代农业加快发展，农业产业化“712”提升行动计划扎实推进。现代服务业统筹发展，商贸物流、文化旅游、现代金融、房地产等产业蓬勃兴起。节能减排目标任务超额完成。

**五年来，城市形象日益彰显。**围绕打造山水园林城市、滨河滨湖城市、宜居宜游宜学宜业城市，统筹推进新区开发、老区提升、矿区改造、景区完善和综合交通。城乡规划体系不断完善，基本实现全覆盖。城市建设蓬勃展开，完成投资492.3亿元，是“十五”时期的7.5倍。竣工商品住房面积775万平方米。新增城市道路面积380万平方米，改扩建农村公路1743公里。高速公路、高速铁路、淮南港和航站楼加快规划建设，综合交通运输能力进一步提高。城市创建深入推进，先后荣获国家园林城市、中国优秀旅游城市、节能减排二十佳城市、资源型城市可持续发展示范市等称号。新增绿地面积586.4万平方米。新创4A级景区2处。新农村建设扎实推进，跻身省级城乡一体化试点市。

**五年来，改革开放深入推进。**实施合淮同城化战略，成为合肥经济圈重要成员，创造性融入皖江城市带承接产业转移示范区，加快融入长三角，加速国际化进程。累计利用外资7.4亿美元，引进内资722.7亿元，实现进出口5.6亿美元，分别比“十五”时期增长83%、675%、210%。加快规划建设11个开发区，省级开发区从1个增加到6个，搭建163平方公里工业园区开发平台。完成36户国有集体企业改革，新增民营企业7500家、个体工商户1万户。深化农村综合改革，完成集体林权制度主体改革。积极推进政府机构改革，强化城乡规划建设、招商引资、沉陷区治理等服务职能。加大行政审批制度改革力度。深入推进事业单位改革。加强创新体系建设。建成国家级研发平台3个，鉴定科技成果167项，荣获各类科技进步奖51项。第六次荣膺全国科技进步先进市。

**五年来，人民生活明显改善。**城镇居民人均可支配收入和农民人均纯收入分别从8599元、2786元增加到15300元、5400元。城乡居民人均住房面积分别从20.9平方米、25.1平方米增加到24.5平方米、35平方米。金融机构存贷款余额分别从333.5亿元、221.9亿元增加到897.8亿元、655.9亿元。投入民生工程资金25.5亿元，惠及240万人。采煤沉陷区综合治理取得重大突破，投入资金60亿元，建设搬迁安置点61个，搬迁居民28.7万人。统筹发展各类教育，全面落实城乡免费义务教育等政策。城乡食品药品安全、公共卫生、医疗服务等体系不断完善。人口计生工作得到加强。累计新增城镇就业17万人，实现下岗再就业11.2万人，转移农村劳动力16万人。社会保障体系进一步完善，累计支出54.5亿元。医疗保险实现“三张网”全覆盖。新型农村养老保险试点工作启动。社会救助830万人次。文体事业丰富多彩，成功举办五届中国豆腐文化节、两届中国**·**淮南国际少儿艺术节、第十二届全省运动会，圆满完成奥运火炬传递。人民群众生活质量和幸福指数明显提高，精神文明和民主法制建设不断加强，安全生产和信访稳定形势平稳，社会保持和谐安宁。

各位代表，五年来，我们风雨同舟、迎难而上，取得了一个又一个胜利；五年来，我们科学发展、奋力赶超，跨上了一个又一个台阶。回首“十一五”，我们深深地感到，要推动淮南新发展、开创新局面、实现新跨越，**必须立足市情、明晰思路。**牢固树立科学的发展观、正确的政绩观、人本的群众观，把中央精神、省里要求和淮南实际结合起来，不断丰富“五彩淮南”的形象定位、“两型城市”的目标定位、“一主两翼”的战略定位、“四煤”发展的转型定位、“四宜”城市的功能定位，闯出资源型城市可持续发展新路。**必须深化改革、开拓创新。**坚持用创新的思维、改革的办法、市场的机制，调动全社会的积极性、主动性、创造性，永不满足、永不停滞、永不僵化，构建转变发展方式体制机制，增强发展活力。**必须统筹兼顾、协调推进**。坚持提升学习力、实践力、执行力，保持整体联动、城乡互动、区域带动，促进经济、社会、文化、生态协调发展。**必须把握当前、放眼长远。**坚持发展不动摇、不折腾、不懈怠，对历史负责、为现实服务、替未来着想。**必须以人为本、改善民生。**坚持问政于民、问需于民、问计于民，把实现好、维护好、发展好人民群众的根本利益作为一切工作的出发点和落脚点，让人民群众共享改革发展成果。

**二、奋发有为、异彩纷呈的2010年**

过去的一年，是国际国内宏观环境极为复杂的一年，也是我市大事多、喜事多的一年，全市经济社会呈现增长较快、结构优化、效益提高、民生改善的良好态势。预计，全市生产总值增长13%以上，财政收入增长41.3%，固定资产投资增长41.6%，社会消费品零售总额增长18.6%，城镇居民人均可支配收入增长8.9%，农民人均纯收入增长10.4%。一年来，主要做了以下工作：

**（一）推进项目招商，在产业培育上迈出新步伐**

以项目投资发展产业。全年实施项目1280个，增长35.9%。扎实推进“工业结构调整年”。中石化（安徽）煤化工基地批准筹建，中安联合煤业化工有限公司挂牌成立。煤制烯烃及乙二醇、郑煤机舜立机械、顾桥煤矸石电厂等项目开工。高倍聚光太阳能发电系统及组件、轻纺服装鞋帽工业城、年产5万辆新能源专用汽车生产线等项目成功签约并启动实施。煤制气、3座电厂、2条1000千伏特高压、毛集博胜太阳能光伏发电等项目前期加快推进。3大煤矿矿建工程全面展开。比塞洛斯煤机、长壁煤机、海螺水泥一期等项目竣工投产。全年净增规模以上工业企业182家。市经济技术开发区“倍增计划”全面完成，凯盛重工等15个项目开工建设，德普胶带等13个项目竣工投产。高新技术产业开发区“三纵两横”路网全面拉开，台湾科技园正式开工，国家煤化工产品质检中心主体工程完工。

以招商引资嫁接产业。制定承接产业转移投资若干优惠政策，建立招商引资重大项目落地推进机制。组建了驻深圳、厦门、杭州招商联络处和274个招商小分队。组团参加了第六届徽商大会、世博安徽活动周等重大节会，扎实开展赴南美、欧洲、香港、长三角等地招商推介。编制了皖江城市带承接产业转移淮南集中区30平方公里规划，与上海杨浦区签订产业发展战略合作框架协议，与上海长宁区、贵州遵义市缔结友好城区（市），与浙江平湖市合作加快，与淮南矿业签订共同推进城市化、工业化框架协议。与央企合作进展顺利，淮南电网工程等9个项目开工，新增签约项目7个。大力实施民营经济发展“4511”腾飞计划，与全国知名民营企业签约项目152个，总投资619亿元。共同建立合肥经济圈定期会商机制，完成合肥经济圈城镇体系规划。投资环境不断优化，荣获“2010浙商重点推荐投资城市”、“2010苏商投资中国首选城市”。全年利用外资1.34亿美元，增长11.7%；引进内资260亿元，增长57.6%；实现外贸进出口总额1.32亿美元，增长10%。

**（二）展现五彩魅力，在城市功能上实现新提升**

“四大”节会成功举办。第八届全国矿山救援技术竞赛，展现了淮南良好的办赛、竞赛水平，为矿山救援事业发展作出了积极贡献。第十七届中国豆腐文化节，坚持以节促发展、以节促开放、以节促招商，取得了文化交流与经贸合作的双丰收。第十届全国“市长杯”乒乓球赛，被国家体育总局赞为“十全十美”的赛事，达到了成功、创新、精彩、难忘的效果。第十二届全省运动会，凸显了“五彩淮南、盛世江淮”的主题，实现了办好一流盛会、建成一流场馆、展示一流形象、务求一流效果、提供一流保障、力争一流成绩“六个一流”的目标。“四大节会”的成功举办，展示了“五彩淮南”的城市形象和辉煌成就，增强了全市人民自信心和自豪感，正转化为加快发展、加速崛起的强大动力。

城市建设全面提速。城市总体规划（2010－2020年）获国务院批准。西部城区分区规划、八公山综合旅游服务区规划、高铁站周边地块设计、滨瓦埠湖新城概念性规划等编制完成，滨河新城、滨高塘湖新城、大汉城等概念性规划启动。全年完成大建设投资221亿元，增长43.8%。**新区开发全面展开。**市级机关办公楼和南纬一路等6条道路基本完工，奥林匹克公园、淮南二中、山南污水处理厂等项目加快推进，志高文化科技动漫产业园、D水系等45个项目全面开工。**老区提升取得突破。**老城区市政基础设施提升三年计划提前实施，硬化、绿化、亮化、净化、智能化和文化“六位一体”推进。国庆路等16条主干道、陈洞路过街人行天桥和广场路下穿立交竣工投用，12条次干道、主城区113条小街小巷改扩建工程完工。拆除违法违规违章建筑27万平方米。**矿区改造扎实推进。**泉大资源枯竭矿区环境修复与开发加快实施，棚户区改造力度加大，改造面积367万平方米。**景区完善再添活力。**谋划旅游项目81个，总投资588亿元。焦岗湖度假村、十涧湖国家城市湿地公园等38个项目加快建设。创建全国绿化模范城市、省级文明城市取得实质性进展。植树造林1.3万亩。上窑国家森林公园被评为国家生态文明教育基地，大通万人坑教育馆、龙湖公园创牌升级扎实推进。**综合交通加快构建。**骆岗机场淮南城市候机楼开业，淮蚌高速公路主体工程完工，206国道改线工程、东西部第二通道、京福高铁淮南段加快建设，淮南东站正式开工。

服务业加快发展。落实扩大消费政策，家电以旧换新、汽车摩托车下乡等活动扎实开展。全年新开工房地产面积629万平方米，竣工面积210万平方米，分别增长166%、30%。淮南月星环球商业中心等项目开工，龙湖路地下商业街等8个项目加快推进。316家企业签订融资协议174亿元。招商银行淮南分行、淮河农村商业银行、凤台通商村镇银行挂牌成立，海通证券进驻淮南。筹建小额贷款公司5家、新批准开业11家。实现旅游收入23亿元，增长15%。

**（三）强化“三农”工作，在城乡统筹上取得新进展**

现代农业加快发展。全面落实强农惠农政策，投入“三农”资金18亿元，增长28.6%。农产品加工业实现产值100亿元，增长23%。新增全国绿色食品原料基地1个、国家农业标准化示范区1个、省级农产品标准化示范基地3个，认证无公害农产品、绿色食品、有机食品22个。新增省级农业产业化龙头企业8家、农民专业合作社110家。凤台县成功申报“中国糯米之乡”地理标志。省级潘集现代农业生态综合开发园区开工建设。

新型农村扎实推进。出台关于开展城乡一体化综合配套改革

的决定，完成城乡一体化总体规划和9个专项方案编制。新农村建设“千村百镇”、“1860”示范工程和“阳光村务”工程稳步实施。上窑洛河一体化、舜耕安成滨河型城镇建设步伐加快。创建省级生态乡镇9个、省级生态村14个。土地流转服务平台加快建设，累计流转土地15.5万亩。启动34.7万亩农村土地整治重大项目，报批各类建设用地1.47万亩。加强土地开发整理，新增耕地1.96万亩。荣获全省耕地保护工作先进市。

农民生产生活条件得到改善。石姚段、洛河洼行洪区退建加固、泥河泵站应急维修一期、一批行蓄洪区撤退路和堵口复堤工程竣工，朱集灌区节水等工程加快推进。新建农村公路75公里，改扩建农村客运站7个。解决11.65万农村人口饮水安全问题。“清洁乡村、美化家园”试点村扩大到122个。建设农村户用沼气池2400口、联户沼气工程3处。新建5座村镇生态式生活污水处理站、7座村镇压缩式垃圾转运站。完成退网还湖1.5万亩。

**（四）深化改革创新，在环境优化上呈现新变化**

各项改革步伐加快。国家资源型城市可持续发展试点总体方案待批。企业改革稳步推进，出台了皖淮机械厂改革重组、搬迁重建总体方案，完成1户中小企业股改。加大向扩权强镇试点镇放权力度，下放管理权限23项。市旅游投资公司挂牌成立。

科技创新步伐加快。抢抓国家技术创新工程试点省建设机遇，5个项目列入试点范围。深部煤炭开采与环境保护国家重点实验室、煤矿生态环境保护国家工程实验室相继获批。获国家科技进步奖2项、国家创新基金项目9个、省级以上创新试点企业5家。认定高新技术企业64家、高新技术产品28个。专利申请1300件、专利授权635件。荣获全省质量兴市优秀等次。毛集被批准为国家科技富民强县试点区。谢家集被批准为省级科技特派员试点区。

节能减排步伐加快。实施500万元以上技术改造项目200个。22项节能和资源综合利用项目深入推进，19个污染减排项目全部建成。拆除机立窑水泥生产线2条，关闭小煤矿2座，关停粘土砖瓦窑厂53座。全年万元GDP能耗下降5%，化学需氧量削减2.14%，二氧化硫排放量低于控制指标8个百分点。

**（五）加强社会建设，在民生改善上得到新拓展**

公共服务不断完善。投入资金10.6亿元，全面完成42项民生工程。新开工采煤沉陷区村庄搬迁安置点12个、续建10个、竣工11个，搬迁居民4.3万人。顾桥首采区村庄搬迁安置应急工程加快实施。中小学校舍安全加固工程扎实推进，竣工面积58.6万平方米。高中阶段教育毛入学率87.4%，高考升学率62.3%。基层医药卫生体制综合改革深入推进，目录类药品全部实行“零差率”销售。新型农村合作医疗实现市级统筹，城镇职工医保、城镇居民医保参加人数分别达47.5万人、59万人。新建标准化村卫生室426个、社区卫生服务中心5个、社区卫生服务站38个。荣获国家文化“群星奖”1项，电视剧《念恩桥》开拍，淮南子文化发掘利用取得新成果。文化体制改革迈出实质性步伐，6家单位完成转企改革。建成乡镇文化站16个、农家书屋100个。我市体育健儿在安徽省第十二届运动会上取得全省第二的优异成绩，并荣获体育道德风尚奖。建成农民体育健身工程54个。全市新型农村社会养老保险试行办法出台并在潘集试点，凤台县进入全国新型农村养老保险试点。发放社会救助金2.1亿元，救助147.5万人次。新建、改扩建农村敬老院10所，农村五保集中供养率达38%。利用住房公积金贷款支持保障性住房建设试点市获批，发放住房公积金贷款16.2亿元，当年使用率提高32%。建成经济适用房30.3万平方米、廉租住房9.8万平方米，发放住房租赁补贴2410.5万元。

社会管理得到加强。新增城镇就业岗位3.9万个，城镇登记失业率4.37%。实现下岗失业人员再就业1.8万人，转移农村劳动力3.6万人。新建农民工创业园9个。淮南被批准为省级创业型城市。第六次人口普查扎实开展。人口低生育水平得到有效控制。深入推进“三项”建设和“三项”重点工作，积极解决社会矛盾，严厉打击各类刑事犯罪活动。信访形势持续好转，进京、赴省、来市上访总量下降。切实加大安全隐患排查和治理力度，安全生产形势总体稳定。应急管理体制不断完善。民族宗教工作继续巩固，国防动员、人民防空和双拥工作继续加强，妇女儿童、老年人、残疾人权益得到保障，地方志、档案、地震、气象等事业取得新发展。

**（六）加强政府自身建设，在管理创新上展示新作为**

大力弘扬“五加二、白加黑”精神，切实提高政府公信力、

执行力和创新力。推进行政决策机制试点市建设，实行人大代表、政协委员列席市规委会制度。深入推进问计于民制度化、常态化，不断拓宽问政于民的新渠道。扎实开展“十二五”规划编制。加强政府立法，提请市人大常委会审议地方性法规5件，制定政府规章3件。办理人大代表议案、建议121件，政协委员提案421件。继续深化行政审批制度改革，推进“一窗式”服务，审批效率大幅提升。强化“五位一体”大督查，促进闭环管理。严格落实党风廉政建设责任制，开展工程建设领域专项治理，强化行政监察和审计监督，坚决纠正损害群众利益的行为，树立了清正廉洁的良好形象。

在肯定成绩的同时，我们还应看到前进道路上存在的困难和问题。主要表现为：经济结构、产业结构、区域结构战略性调整需要加快，非公经济、园区经济、县区经济发展还不充分，服务业、高新技术产业比重偏低，综合竞争力有待提升；农民增收的基础还不稳固，“三农”工作仍需强化；转变发展方式要求迫切，生态治理、环境修复、节能减排和耕地保护任务艰巨；城市管理水平不高，拆违控违力度仍需加大；改革开放需要深化，创新能力有待提高；安全生产不容忽视，维护稳定压力较大；政府职能转变还不到位，公共服务有待加强，发展环境仍需优化。对此，我们将高度重视，采取切实措施，认真加以解决。

各位代表，“十一五”取得的成绩来之不易，积累的经验弥足珍贵，创造的财富影响深远。这是省委、省政府和市委坚强领导的结果，是市人大、市政协关心支持的结果，是全市人民奋发进取的结果。这里，我代表市人民政府，向全市人民，向驻淮部队、武警官兵和政法干警，向各民主党派、各人民团体和社会各界人士，向关心支持淮南现代化建设的驻淮单位和海内外朋友，

向在淮创业的投资者和建设者，表示衷心地感谢！

**三、加速崛起、全面转型的“十二五”**

未来五年，是淮南全面建设小康社会的关键时期，也是构建“两型城市”的起步阶段，更是推进“一主两翼”的加速时期。随着经济全球化的深入发展、国家区域战略的全面实施、东部产业的梯次转移、安徽经济的加速崛起、全市集聚能量的陆续释放，淮南正迎来跨越发展的大好机遇。我们一定要立足新起点，把握新形势，顺应新期待，投身新实践，共同开创“十二五”发展的美好未来。

根据市委八届十三次全会《建议》，“十二五”时期经济社会发展的指导思想是：以邓小平理论和“三个代表”重要思想为指导，深入贯彻落实科学发展观，以转变发展方式、加速崛起进程、实现富民强市为主线，以建设“两型城市”为着力点，以“四煤”发展为主要路径，以扩大开放和改革创新为动力，以保障和改善民生为落脚点，大力实施新型工业化、新型城市化、城乡一体化、合淮同城化、创新推动、可持续发展“六大”战略，推进转型发展、绿色发展、开放发展、安全发展、和谐发展，打造依山傍水、滨河滨湖、宜居宜游宜学宜业的淮河流域现代化中心城市。

“十二五”时期经济社会发展的主要目标是：到“十二五”末，实现三个翻番、四个提高。

**三个翻番：**即实现经济总量翻番，地区生产总值达1270亿元以上，进入千亿元城市行列；财政收入翻番，达212亿元；城镇居民人均可支配收入和农民人均纯收入翻番，分别达3万元和1万元。

**四个提高：**即工业化水平明显提高，工业增加值达750亿元；城市化水平明显提高，城市化率达70%，社会消费品零售总额达400亿元；生态环境质量明显提高，生态体系建设取得显著成效，节能减排完成控制指标，工业固体废弃物综合利用率达95%；群众幸福感明显提高，城镇登记失业率控制在4.3%以内，高中阶段教育毛入学率达91%，学前三年教育毛入园率达75%，人口自然增长率控制在8.5‰以内，城乡公共服务逐步实现均等化，人民生活加速改善，社会保持和谐稳定。

“十二五”时期的主要战略任务是：

**（一）坚持转型发展，加速新型工业化。**推进转型发展，关键要把经济增长转到以现代农业为基础、战略性新兴产业为先导、先进制造业和现代服务业为支撑的发展轨道上来，培育“两型”产业，发展“两型”经济。**坚持把立足煤作为转型发展的动力之基。**加强煤炭清洁高效利用，构建安全、稳定、经济的现代能源产业体系，打造千亿级煤炭产业群、五百亿级电力产业群。大力发展循环经济，提高煤炭伴生资源综合利用率。**坚持把延伸煤作为转型发展的动力之源。**依托先进煤化工储备技术、煤化工产业园、大型煤化工企业，加强煤气化联合循环发电技术、以煤气化为核心的化学品多联产系统、合成油多联产系统的研究和应用，推进煤—电—化一体化、煤—电—铝一体化、煤—电—硅—光伏一体化，打造双百亿级煤化工产业群。**坚持把不唯煤作为转型发展的重中之重。**提升煤机产业的集聚度、关联度和耦合度，建成国内知名的煤机成套生产线，打造双百亿级装备制造产业群。加快新能源、新材料、生物医药、节能环保等战略性新兴产业培育，建设太阳能光伏生产基地、特种工程运输车辆制造基地、生物医药及高新技术产业基地，打造双百亿级汽车及零配件产业群、5个50亿级产业群。完善现代农业产业体系，建设十大优势农产品基地、五大现代农业示范区，实施“吨粮市”和新一轮“菜篮子”两大工程，打造现代农业加工物流园区、豆制品加工物流园区、农业投资公司三大平台，培育市级龙头企业100家、农民专业合作组织500家，打造沿淮粮仓和省级绿色食品加工基地。**坚持把超越煤作为转型发展的不懈追求。**以城市大建设推动服务业大提升，加快发展文化、旅游、商贸、物流、房地产等生活性服务业，着力壮大金融、保险、科技等生产性服务业，拓展新领域，发展新业态，培育新热点，打造一批现代服务业集聚区。着力提升商贸物流业，增强龙湖路商业中心、西部商业中心功能，规划建设山南新区商业中心，做大做强一批物流园区。着力提升文化旅游产业，打好淮南子文化、花鼓灯文化、少儿文化、豆腐文化和八公山旅游文化，实施千亿文化旅游振兴工程，打造文化之都。着力发展现代金融业，推进金融产品和服务创新，规划建设山南金融集中区，推进中小企业信用体系试验区建设。加强信息资源整合，积极发展物联网。以国家技术创新工程试点省建设为契机，完善以企业为主体、市场为导向、政银产学研相结合的技术创新体系，打造“四点一园”自主创新高地。高新技术产业增加值占全市生产总值15%以上。到2015年，形成销售收入超千亿企业1户、超百亿企业10户、超十亿企业30户、超亿元企业100户以上。

**（二）坚持绿色发展，推进新型城市化。**把优化空间布局、提升城市能级、彰显五彩特色作为城市化的主要任务，推进“四规合一”，努力建设淮河明珠。**打造滨河滨湖城市。**实施跨河联动发展，推进淮河治理与主城区、北部新区一体化开发，加快滨河新城建设。推进高塘湖与东部新区、上窑古镇、交通枢纽一体化开发，加快滨高塘湖新城建设。推进瓦埠湖与西部新城区、旅游服务区一体化开发，加快滨瓦埠湖新城建设。推进凤凰湖与采煤沉陷搬迁安置区一体化开发，加快滨凤凰湖新区建设。推进毛集景区、园区、城区互动发展，加快滨焦岗湖生态新区建设。**打造山水园林城市。**树立生态文明理念，坚持绿色发展、低碳发展。以创建国家生态园林城市、全国绿化模范城市、国家卫生城市、全国文明城市为抓手，推进“四城联创”。坚持保护与开发并重，推进八公山与西部城区、舜耕山与山南新区、上窑山与东部新区一体化开发。实施“十大”林业工程，构建“六大”绿色体系。加强节能减排和环境保护，推进城乡环保一体化。到2015年，建成区绿化率达45%以上、绿地率达38%以上、人均公共绿地面积15平方米以上，森林覆盖率达22%以上。**打造“四宜”城市。**以宜居为本，建设原生态的新区、现代化的老区、人本化的矿区。以宜游为要，念好山水经、打好文化牌、做好无中生有文章，建好能源城中的旅游城、旅游城中的能源城。以宜学为先，加快建设职教园区，促进优质资源共建共享，打造学习型城市。以宜业为重，构建立体交通，发展综合运输。到2015年，城市建设投资超过2000亿元。

**（三）坚持开放发展，开创合淮同城化新局面。**把握新一轮对外开放前沿阵地的战略机遇，在更大范围利用两个市场、两种资源。**以开放促进市场化改革。**加大改革攻坚力度，进一步理顺政府与企业、市场、社会的关系，创造更具活力的体制环境。继续深化国有企业改革，引导非公企业、民营企业发展。理顺财政分配关系，完善有利于产业结构升级、服务业提升和县区跨越发展的税收政策。做好资源性产品价格改革，建立生态补偿和资源补偿机制。**以开放提升同城化速度。**把合淮同城化作为合肥经济圈的强核之举，加强体制机制、基础设施、项目谋划、招商引资、市场准入、政策体系等方面的有效衔接，形成一体化的格局，提升合肥经济圈实力。推进合淮工业走廊建设，努力建成区域合作的引领区、承接转移的示范区、科技创新的先行区、高端产业的集聚区、跨越发展的增长极。**以开放提高“引进来”水平。**把招商引资与承接产业转移、“飞地经济”发展、深化央企省企民企合作、建立友好城市结合起来，打好招商引资“组合拳”。依托园区平台，高效推进招商引资，打造国家级经济技术开发区、安徽（淮南）煤化工产业园、高新技术产业开发区、皖江城市带承接产业转移淮南集中区。**以开放加快“走出去”步伐。**完善支持企业“走出去”的政策和服务体系，提高经营水平和投资效益。完善外贸促进机制，深入实施“外贸孵化器”工程，促进对外贸易转型升级，构建现代营销体系和商务体系。**以开放推动本土化进程。**支持大企业领跑工业化、参与城市化、反哺农业现代化，做大做强总部经济。引导外来企业技术研发、产业配套、管理人才本土化，促进外资企业“落地生根”。调动县区的积极性和创造性，激发内生活力，引导各地走因地制宜、特色发展之路。“十二五”期间，通过利用外资、引进内资、启动民资、扩大融资，确保完成固定资产投资5000亿元，力争6000亿元。

**（四）坚持安全发展，打造平安淮南。**牢固树立“安全为天、生命至上”理念，强化安全发展的政治责任，打造本质安全型城市。**确保淮河安澜。**坚持开发和治理并重，充分发挥淮河优势，加快新一轮治淮工程建设，统筹解决水多、关注水少、改善水脏、发展水运、兴建水景，打造安澜之河。**确保煤矿安全。**坚持“安全第一、预防为主、综合治理”方针，加大煤矿瓦斯防治、火灾防治、水害治理力度。围绕装备现代化、系统自动化、管理信息化，实施人防、技防、物防相结合，打造现代化安全矿井。严格落实安全生产责任制，深入排查和消除安全隐患，强化重点领域安全监管，坚决遏制重特大安全事故发生，实现安全生产形势持续稳定。**确保社会安定。**加强社会治安防控体系建设，深入开展严打专项整治，依法打击各种违法犯罪活动。健全维护群众权益机制，妥善解决人民群众反映强烈的突出问题。完善稳定风险和信访评估制度，构建矛盾纠纷大调解工作体系，推进人民调解、行政调解、司法调解联动。完善抗灾救灾、公共安全、社会安全、食品药品安全等预防预警体系，提升应急处置能力。积极推进民族团结和宗教工作，努力形成社会安定、环境安全、百姓安宁的良好局面。

**（五）坚持和谐发展，拓展城乡一体化空间。**从群众最关心、最直接、最现实的事情做起，按照城乡一体化的新要求，把公共资源更多地投向农村地区、社会事业和民生领域，提高群众的幸福指数。**建设农村幸福美好家园。**围绕农业产业化，统筹县区开发区、农产品加工区和物流园区建设，加快企业向园区集中。围绕农村社区化，统筹推进新农村、新市镇、行蓄洪区和采煤沉陷区村庄搬迁、户籍制度改革，加快人口向城镇集中。围绕农民现代化，统筹整村、整乡和整区推进，加快土地向规模集中。努力实现农业好收成、农民好收入、农村好局面。**打造文化强市。**深化社会主义核心价值体系建设，把文化融入产业培育、城市建设、招商引资和群众生活之中，完善公共文化服务体系，实现经济效益和社会效益有机统一，构建与经济社会发展水平相适应的文化管理体制、运行机制和发展格局。**办好人民满意教育。**坚持教育优先发展、均衡发展、规范发展，把提高质量作为教育的核心任务，加快“学有所教”向“学有优教”转变。继续深化教育改革，积极发展学前教育，着力提升义务教育，全面普及高中教育，加快发展高等教育，支持发展民办教育，建立以就业创业为核心的职业教育和培训体系。实施人才强市战略，完善多元化投入机制，统筹推进各类人才队伍建设。**深化医疗卫生体制改革。**按照保基本、强基层、建机制的要求，整合医疗资源，完善城乡医疗卫生服务体系，普及基本公共卫生服务。大力开展全民健身运动，提高人民群众健康素质。全面做好人口和计划生育工作。**促进就业一体化。**实施更加积极的就业政策，大力发展劳动密集型产业、服务业和小型微型企业，完善一体化公共就业服务体系。**健全城乡社会保障体系。**坚持广覆盖、保基本、多层次、可持续方针，实现城乡社会保障体系、社会救助体系全覆盖。加快发展社会福利和慈善事业，建成市社会福利保障区、市防灾救灾指挥中心等重点工程。**增进人民群众福祉。**深入实施民生工程，着力提高民生工程质量和效益，建立健全民生工程长效机制。坚持把采煤沉陷区综合治理作为最大的民生工程，推进集中式搬迁、发展式安置、开发式治理，让人民群众共享改革发展成果，让淮南的山更绿、水更清、天更蓝、人与自然更加和谐。

**四、乘势而上、大有可为的2011年**

今年是“十二五”的开局之年，做好今年的工作至关重要。主要预期目标是：地区生产总值增长12%以上，财政收入增长15%，固定资产投资增长30%以上，社会消费品零售总额增长17%，城镇居民人均可支配收入力争增长14%，农民人均纯收入力争增长14%，城镇登记失业率控制在4.3%以内，人口自然增长率控制在8‰以内，节能减排完成省下达的目标任务。

为实现上述目标，着力抓好七个方面工作。

**一是推动“两型城市”规划、设计和建设**

加强战略规划。把资源节约型和环境友好型理念，贯穿于经

济、社会、文化、生态建设之中，加强“两型城市”规划，出台总体实施方案和具体实施细则，推进“两型城市”建设。

开展系统设计。坚持高端定位，制定目标体系，突出工作重点，合理有序推进。建立健全导向机制，构建产业发展、对外开放、城矿乡统筹、行政、投融资、科技创新和人才管理等机制。

加快建设步伐。夯实“两型城市”基础，加快实施产业支撑、基础设施建设、生态建设、城矿乡统筹、人才和人力资源开发、示范区建设等六大工程。加快培育“两型”产业结构、增长方式和消费模式。

**二是推动投资、引资和融资突破**

狠抓项目投资。坚持扩大有效投入，全年完成投资520亿元以上。新开工项目169个、续建339个。编制“十二五”投资项目库，储备项目投资规模超过1.2万亿。坚持保障发展与保护耕地并重，加大建设用地报批和调度力度，加快土地整体推进，新增耕地1万亩。建立项目审批“绿色通道”，完善政务服务中心功能，建立三级联动电子政务监察网络。

狠抓招商引资。全面开展“招商引资年”活动，大规模、快节奏、高频率推进招商引资。发挥能源、载体和政策等综合优势，创新“一把手”、联络处、商会和节会等招商方式，精心组织工业、城建、服务业和农业等专题招商。强化招商引资硬约束，全年引进内资增长30%以上，利用外资增长20%以上。办好合肥经济圈第二次会商会，提升开放合作水平。深入有效地推进产业承接，加快“飞地经济”发展，引进一批牵动性强、技术含量高的项目落户淮南。瞄准央企新一轮战略导向，完善合作机制，推动项目滚动发展。落实优化投资环境的实施意见，完善重大项目落地推进工作机制。加强出口基地建设，实现外贸进出口总额增长10%以上。

狠抓市场融资。抓住并用好扩大内需政策，积极争取中央和省级配套资金。加快投融资公司改革和组建步伐，挂牌成立教育、农业投资公司。创新保险、担保、基金等融资方式，五大投资公司实现融资120亿元。创新银政银企对接方式，开展以企业和行业、园区和县区、产业链和供应链为基础的融资对接活动。引进市外金融机构1家，支持通商银行组建村镇银行。筹建小额贷款公司2家，开业2家。支持企业联保贷款。加强担保公司规范化建设，新组建5家。

**三是推动工业、农业和服务业发展**

促进工业提速。工业增加值增长15%以上，力争完成工业投资200亿元。建成高倍聚光太阳能发电系统及组件、顾桥矿技术改造等一批重点项目。加快煤制烯烃及乙二醇、郑煤机舜立机械等重大项目建设。开工平电三期、淮沪二期、淮浙二期、新能源专用汽车生产线等项目。推动朱集东矿、朱集西矿、杨村矿核准开工。做好煤制气项目一期、洛电四期、2条1000千伏特高压、山南新区热电联产等项目开工准备。继续实施七大产业调整振兴规划，出台加快纺织业发展实施方案。大力实施“223”工程，培育新的增长点。

促进农业增效。投入“三农”资金21亿元，增长15%。抓好

100万亩优质小麦、100万亩优质水稻、50万亩特种蔬菜、20万亩绿色大豆等标准化基地建设。出台加强“菜篮子”工程建设的意见。完成78个农贸市场升级改造。培育农产品加工产业集群，推进农业示范园区建设。农产品加工业产值增长20%，达到120亿元。建成省级标准化生产基地2个、优质农产品基地检测点20个，认证无公害产品、绿色食品、有机食品20个。新增农民专业合作社50家。加快“三个集中”，启动10个示范乡镇、50个示范村一体化建设。推进10个新农村示范村建设。完成6个村扶贫开发整村推进任务。新增178个“农村清洁工程”示范点。大力发展劳务经济，培训新型农民1.5万人。累计流转土地面积25万亩。新增退网还湖面积2.7万亩。抢抓新一轮治淮工程建设机遇，完成禹王泵站更新改造等6大工程，实施焦岗湖、港河、架河平原洼地治理项目，力争董峰湖行洪区调整改造、西淝河治理工程、高塘湖排涝泵站等项目批复实施。

促进服务业繁荣。继续推动消费升级，提前实现“双百亿”商贸战略。建成龙湖路地下步行街等7个项目，开工建设信谊国际购物广场等项目。继续推进万村千乡市场工程、家电汽车摩托车下乡工程、家电以旧换新活动，建立覆盖城乡的现代商贸流通一体化网络。开工建设安徽沪华物流园、宝元物流园，建成安成鑫海物流园、医药物流园。加快文化产业发展，开工建设大汉城、淮南子文化产业园、淮南大剧院、市文化馆、市图书馆等项目。推进有线电视数字化改造。办好第十八届中国豆腐文化节、第三届中国**·**淮南国际少儿艺术节。开工建设房屋面积400万平方米以上，竣工面积200万平方米以上。

**四是推动城市规划、建设和管理提升**

统筹城乡规划与项目谋划。完成轨道交通、抗震防灾、上窑风景区、东部地区产城一体化等规划编制。完成主城区20平方公里、山南职教园区、高新技术产业开发区等控制性详细规划。完善村镇规划。建立以规划生成项目机制。力争完成城市建设投资400亿元。

统筹城市功能定位与基础设施建设。推动新区向宜居宜业跨越，以更大力度推进山南新区起步区15平方公里开发，支持淮南矿业参与山南新区建设，启动13.6平方公里开发。建成淮南二中、农民安置新村等12个项目，推进山南城市综合体、正源大厦等37个项目，开工建设市会展中心、五星级酒店等52个项目。推动老区向滨河滨湖跨越，以更高标准启动滨河新城、滨高塘湖新区建设，开工建设谢三村街道改造和16个老城区综合改造项目。整治城市旧小区30个。推动矿区向环境友好、安全和谐跨越，改造棚户区320万平方米、竣工122万平方米。推动景区向区域联动、旅城一体跨越，提升八公山、上窑、焦岗湖、茅仙洞风景区水平，建成龙湖公园二期，力争志高文化科技动漫产业园开园，加快建设焦岗湖国家湿地公园核心区等20个项目，开工建设舜耕山风景区、淮河公园、淮西湖公园，规划建设山南旅游主题区、春申君及楚文化旅游区、现代农业休闲观光旅游区、淮河民俗风情带。

统筹立体化交通与精细化管理。对外发展综合运输，建成淮蚌高速公路，做好济祁、淮滁高速公路前期工作；建成淮南东站暨站前广场，推进商杭高铁前期服务工作。加快孔李淮河大桥建设，开工建设淮河隧道、淮南新港；规划新桥机场淮南航站楼，开工建设淮南大道、九龙大道和山南新区快速路网。对内构建立体交通。制定加快交通基础设施建设意见。优化县区路网和公交线路。启动智能交通安全系统建设。开工滨湖大道等7个新建工程、朝阳西路等12个改扩建工程、电厂路等3个下穿立交工程，推进卧龙山路等9个续建工程、国庆路等6条道路亮化工程、城市防洪排涝工程，建成东西部第二通道等15条道路。加快创建省级文明城市、全国绿化模范城市。理顺城市管理体制，创新城市管理机制。推进以公司化运作为核心的环卫市场化管理机制，建立以网格化为基础的绩效管理机制，实施以信息化为支撑的城市联动管理机制，落实以规划为重点的依法管理机制。提高环卫作业机械化水平，开展城管志愿者服务活动。强力拆除和制止违法违规违章建设，建立监管长效机制。加大城市交通管理力度，集中整治乱停乱放、乱穿乱行、乱涂乱画等行为。加强新型社区建设，完善社区功能。成片造林1.3万亩，新增城市绿地100万平方米。

**五是推动县区、园区和非公经济壮大**

夯实富民强县强区基础。加快建设东部城区特色工业基地、商贸物流基地、现代服务业基地，打造产城一体化样板区。支持西部城区装备制造、现代商贸、旅游文化、现代农业发展，打造滨湖新城。支持凤台非煤产业、非公经济发展，积极争创全国现代农业示范县，提升在中部百强县、安徽十强县中的地位，发挥城市副中心辐射带动作用。出台加快北部新区发展的意见，支持能源配套、资源综合利用等产业做大做强，建设新型煤电化主战场。发挥毛集政策优势、创新优势，加快生态旅游、特色农业和新型工业发展。

培育新型园区增长极。发挥园区对外开放“主阵地”作用，完善园区“八通一平”，推进产城一体、功能集聚和扩容升级。实施市经济技术开发区提升战略，主要经济指标均增长20%以上。开工建设轻纺服装鞋帽工业城等17个项目，加快建设淮南月星环球商业中心等20个项目，确保北新建材等19个项目竣工投产。加快安徽（淮南）煤化工产业园、高新技术产业开发区、皖江城市带承接产业转移淮南集中区建设，分别完成投资20亿元、15亿元、5亿元，共引进资金20亿元以上。推进40户企业退城进园。支持县区工业园区和乡镇工业集聚区建设。

促进非公经济发展。认真落实非公经济发展新36条，继续实施民营经济发展“4511”腾飞计划。深入推进与全国知名民营企业合作发展，建立中小企业公共服务平台，对民营企业实行动态支持。积极培育省级专业集群镇。全年新增民营企业1000户以上、个体工商户3500户以上，非公经济增加值增长16%以上。

**六是推动改革、创新和环境优化**

增强改革活力。争取国家资源型城市可持续发展试点。健全国有企业法人治理结构，规范“出中心”企业资产管理。深化建安总公司等3户企业改革重组，再培育3户上市辅导企业。启动国有企业职工债权清偿等工作。推进基层医药卫生体制改革，探索公立医院改革。统筹行政管理体制改革，深化事业单位改革。启动交通投融资体制改革。加快潘集户籍制度改革。

增强创新动力。争创全国科技进步先进市，加快毛集国家可持续发展先进示范区建设，争取潘集批准为省级可持续发展实验区。大力发展实验室经济。探索在开发区设立产业技术研究院。创建国家防爆电气优质产品生产示范区，启动深部煤炭开采与环境保护国家重点实验室、淮南科技创新公共服务中心建设，培育省级企业技术中心4家。申报省级高新技术企业12家、省级创新型试点企业3家、省级高新技术产品30项，高新技术产业实现增加值15亿元，增长50%。新增省级名牌4个、商标16件，力争实现国家级名牌、商标零的突破。主导制定国家标准2项。加大知识产权保护力度，申请专利1500件以上。

增强环境引力。坚持发展与治理兼顾、疏导与防堵结合、增优与减劣并重，加大改造、限制和淘汰力度，确保完成节能减排目标任务。推进合同能源管理，认定资源综合利用企业20家以上。启动火电行业机组脱硝工作。开工建设潘集区、毛集实验区、上窑镇等污水处理厂，拓展西部和凤台县污水管网，建成山南污水处理厂。推进城乡环保一体化，开工5个村镇生态式生活污水处理工程。创建省级生态乡镇1个、省级生态村3个。

**七是推动民生、社会和平安建设**

着力改善民生。实施42项民生工程，其中省级33项，保留市级8项，新增农房改造和城市旧小区综合整治1项。积极推广采煤沉陷区综合治理“后湖模式”、“鑫森物流模式”，创新凤凰湖综合安置模式。强化企业和政府责任，完善地矿协调长效机制，设立综合治理发展专项资金。加大采煤沉陷村庄征地拆迁安置力度，推进应急工程建设，新开工采煤沉陷区村庄搬迁安置点10个、竣工8个。推进住房公积金贷款支持保障性住房建设试点，完成廉租住房6万平方米，新开工公共租赁住房4万平方米。继续推进农村安全饮水工程。完成26个乡镇文化站、251个农家书屋建设。全面加强价格调控监管，保持价格基本稳定，保障市场供应。

着力发展教育。全面实施教育改革和发展规划纲要。组织实施学前教育三年行动计划。继续推进义务教育均衡发展，加强标准化和信息化建设，完成中小学校舍安全加固工程65.8万平方米，重建校舍12.8万平方米，建成市青少年校外活动中心。支持高等教育发展，开工建设安徽理工大学新校区、淮南技师学院。支持职业教育、特殊教育和民族���育发展。继续实施“人才青苗工程”。新建1个国家级和2个省级企业博士后科研工作站。

着力完善医疗卫生服务体系。建立基本药物优先选择、合理使用机制，全面推进“规范药房”建设。规划建设市立医院、市精神卫生中心、市职业病防治院等7所医疗机构，完成41个社区卫生服务机构规范化建设。建成全市农村卫生室信息网，实现新型农村合作医疗门诊统筹“一卡通”。巩固城镇基本医疗保险全覆盖成果，实现职工医疗保险市级统筹。建立医疗救助“一站式”服务平台。统筹人口计生工作，提高人口素质。

着力扩大就业和社会保障。构建城乡一体化就业平台，新增

城镇就业3万人、转移农村劳动力3万人、下岗失业人员再就业1.7万人。加快创业园和公共就业实训基地建设，完善全民创业服务体系。强化欠薪防范和处置，切实保障劳动者合法权益。全面落实社会保险关系转移接续政策，加强扩面提标，完成“五险合一”154.2万人。扩大新型农村养老保险制度试点范围，建立非就业城镇居民养老保险制度。进一步健全社会救助体系。加快市老年公寓建设，建成市光荣院。落实五保政策，集中供养率达45%。完成第八届村民委员会换届选举工作。

着力维护社会和谐稳定。完善应急管理体制机制和预案体系，提高保障公共安全和处置突发事件能力。加强社会治安综合治理，深入推进“打击犯罪新机制”建设。持续开展大接访活动，努力做好信访稳定工作。加强重大危险源监控、重大安全隐患整改和安全生产专项整治，依法治理超载超限，防止重特大事故发生。加强食品药品监督、工商、质监等工作，提高市场监管水平。加强国防动员和民兵预备役工作，密切军政军民关系，争创全国“双拥”模范城市。高度重视妇女儿童、残疾人和老龄工作，全面发展外事、侨务、地震、气象、档案、地方志等事业。

各位代表，完成未来五年战略任务，实现“十二五”精彩开局，需要全市上下共同努力，需要政府主动作为。我们必须志存高远、勇挑重担、破浪前行，推动各项工作提速、提质、提效。**一要增强科学发展、跨越发展的能力。**面对转变方式、优化结构的艰巨任务，唯有科学发展，才能全面转型。面对你追我赶、竞相发展的竞争态势，唯有跨越发展，才能富民强市。我们必须加强学习，提高驾驭复杂局面的能力、解决实际问题的能力、深化改革创新的能力，增强工作的预见性，把握工作的主动权，提高工作的执行力。必须科学决策，使各项政策更加符合淮南实际，经得起历史检验。必须依法行政，加强和改进政府立法，推动管理型政府向服务型政府转变。必须求真务实，树立“干不到一流就是失职、争不到先进就是落后”的进取意识，提升标杆，争先进位，勇攀高峰。**二要提高改善民生、维护民利的本领。**要坚持群众观点，站稳群众立场，健全群众工作制度，切实加强网络问政，始终与人民群众同呼吸、共命运、心连心。要坚持群众路线，想问题、做决策、干工作，都要从群众利益出发，持之以恒地为群众办实事、做好事、解难事。要坚持群众方法，自觉深入困难最集中、矛盾最复杂、群众最需要的地方，认真解决企业改制、征地拆迁、环境保护、劳动争议、涉法涉诉等领域的突出问题，增强做好新形势下群众工作的本领，保障人民群众的合法权益。**三要秉持“五加二、白加黑”的精神。**要始终保持奋发有为、只争朝夕的工作状态。要切实增强干事创业的主动性、创造性，真正把心思花在谋发展、抓发展上，把精力用到干实事、求实效上。要建立健全激励机制、轮岗交流机制，实施民主评议科长活动，让能干事者有机会、会干事者有舞台、干成事者有地位，培养任劳任怨、无怨无悔的职业精神。**四要树立风清气正、高效廉洁的形象。**要自觉接受人大、政协、舆论和社会监督，重视司法监督，加强审计监督，认真听取民主党派、工商联、无党派人士和各人民团体的意见。要全面推行政务公开，让权力在阳光下运行。要切实落实责任制和责任追究制，对失职渎职、不作为和乱作为的，严格行政问责。要厉行节约，规范公务接待，从严控制公费出国出境。要认真贯彻《廉政准则》，落实党风廉政建设责任制，坚决查处违法违纪行为，始终保持为民、务实、清廉的优良作风，树立勤政、廉政、优政的良好形象。

各位代表，新的任务光荣艰巨，新的目标催人奋进。让我们紧密团结在以胡锦涛同志为总书记的党中央周围，在市委的坚强领导下，凝聚全市人民的智慧和力量，坚定信心，奋发图强，努力把“十二五”规划的宏伟蓝图变成跨越发展的美好现实，奋力谱写全面建设小康社会的崭新篇章！
